# Strain-induced density fluctuations in linear low-density polyethyl­ene

**DOI:** 10.1107/S1600576725002183

**Published:** 2025-04-04

**Authors:** Mizuki Kishimoto, Kazuki Mita, Hiroki Ogawa, Kazuki Shibasaki, Masato Arakawa, Mikihito Takenaka

**Affiliations:** a Institute for Chemical Research Kyoto University Gokasho, Uji Kyoto611-0011 Japan; b Mitsui Chemicals 580-32 Nagaura Sodegaura Chiba299-0265 Japan; Argonne National Laboratory, USA

**Keywords:** density fluctuations, mechanical melting, X-ray scattering, linear low-density polyethyl­ene, spatial homogeneity

## Abstract

In our previous study [Kishimoto *et al.* (2020). *Macromolecules*, **53**, 9097–9107], density fluctuations were induced on the submicrometre scale in polyethyl­ene (PE) during strain and led to the generation of voids. The strength of the induction was found to increase with the amplitude of the spatial inhomogeneity of the stress field. In this work, we investigated how comonomer species and content affect the behaviors of the strain-induced density fluctuations in linear low-density PE. We found that the strength of the induction decreased with an increase in the spatial homogeneity before stretching and the mechanical melting during strain and that the degree of spatial homogeneity and mechanical melting depend on the species and content of the comonomer.

## Abstract 2

This study investigated how comonomer species and content affect strain-induced density fluctuations in linear low-density polyethyl­ene. It was found that increased spatial homogeneity before stretching, influenced by the type and amount of comonomer, suppressed the strength of density fluctuation induction during strain.

## 1. Introduction

Linear low-density polyethyl­ene (LLDPE) is used widely in our daily life owing to its excellent properties such as high tensile strength, high impact resistance, flexibility and so on. LLDPE is made by copolymerization of ethyl­ene and α-olefins. Various LLDPEs with different comonomer characteristics such as species and content have been produced industrially. LLDPE exhibits various mechanical properties depending on the comonomer characteristics, and hence we can select the LLDPE suitable to our purpose. The variety of the mechanical properties originates from the change in the crystalline structure of the LLDPE with the comonomer characteristics. It is well known that crystalline polymers form hierarchical structures including crystalline lattice structures, lamellar structures and spherulitic structures filled with lamellar branching structures. To understand the effects of the comonomer characteristics on the mechanical properties, we need to investigate the change in the hierarchical structures of the LLDPEs with different comonomer characteristics under strain.

In our previous paper, we investigated how the hierarchical structures affect the mechanical properties of high-density polyethyl­ene (HDPE) and LLDPE during uniaxial stretching using time-resolved X-ray scattering methods (Kishimoto *et al.*, 2020 ▸) and scanning transmission X-ray microscopy (Arakawa *et al.*, 2022 ▸). Their scattering patterns in the ultra-small-angle X-ray scattering (USAXS) region showed so-called abnormal butterfly patterns after some extent of strain, indicating that the enhancement of the density fluctuations on the order of 100 nm to 1 µm (submicrometre scale) was induced by strain. Further strain was found to cause transformation of the density fluctuations into voids. The enhancement or strain-induced density fluctuation (SIDF) originates from the viscoelastic effect found in dynamically asymmetric systems such as gel under strain and semidilute polymer solution under shear (Bastide *et al.*, 1990 ▸; Mendes *et al.*, 1996 ▸; Kume *et al.*, 1997*a* ▸; Kume *et al.*, 1997*b* ▸; Moses *et al.*, 1994 ▸; Hashimoto & Kume, 1992 ▸). In these systems, concentration or density fluctuations induce the spatial inhomogeneity of the stress fields and the gradient of the stress fields enhances the spatial inhomogeneity of the concentration or density fluctuations under deformation (Doi & Onuki, 1992 ▸; Furukawa & Tanaka, 2009 ▸; Kurotani & Tanaka, 2022 ▸). In the cases of HDPE and LLDPE, the spatial inhomogeneity of the stress field originating from the spatial inhomogeneity of the crystallinity exists as an initial state and the gradient of the stress fields enhances the spatial inhomogeneity of the crystallinity under uniaxial strain. The strength of the SIDF in the LLDPE was found to be weaker than that in the HDPE, reflecting that the spatial inhomogeneity of the stress field of the LLDPE was smaller than that of the HDPE. According to the theory of the viscoelastic effects (Doi & Onuki, 1992 ▸; Furukawa & Tanaka, 2009 ▸; Kurotani & Tanaka, 2022 ▸), the driving force of the SIDF is proportional to the gradient of the stress field. Thus, if the melting of crystalline structures that occurs is associated with the application of the strain, the spatial inhomogeneity and the gradient of the stress field decrease and the SIDF is suppressed. The melting of the crystalline phase in the LLDPE associated with the application of the strain was found to be more marked than that in the HDPE. The spatial inhomogeneity of the stress field and the SIDF in the LLDPE are thus less pronounced than those in the HDPE. Note that a number of deformation models below the 100 nm scale have been proposed based on time-resolved small-angle (SAXS) and wide-angle (WAXS) X-ray scattering measurements. Some of the models were mentioned in the introduction of the previous paper (Kishimoto *et al.*, 2020 ▸).

It is well known that the stability of LLPDE crystalline structures depends on the comonomer characteristics (Shirayama *et al.*, 1972 ▸; Simanke *et al.*, 2001 ▸). We anticipate that the SIDF behaviors in LLDPE are also affected by the comonomer characteristics. In this study, we thus investigate how the comonomer characteristics affect the SIDF behaviors and clarify the factors governing the SIDF in the comonomer characteristics of the LLDPE.

## 2. Materials and methods

### 2.1. Sample characterization

Four types of LLDPE shown in Table 1 ▸ were used. The sample names reflect the comonomer species and the crystallinity of the sheets as characterized by the following experimental procedure. Note that MP-60 is the same as the LLDPE in our previous study (Kishimoto *et al.*, 2020 ▸).

A 30 wt% LLDPE/*o*-di­chloro­benzene-d_4_ solution was prepared for the four types of LLDPE. Pulsed Fourier transform ^13^C nuclear magnetic resonance (FT-^13^C-NMR) measurements were performed at 130°C using a Bruker Avance III 600 MHz spectrometer. The proton decoupling was achieved only during acquisition to suppress the nuclear Overhauser effect. Because the longest spin-lattice relaxation time *T*_1_ in the LLDPE was 3.6 s, derived from methyl carbons, the delay time between pulses was set to 20 s (>5*T*_1_). The comonomer species and content were determined by the method of Pooter *et al.* (1991 ▸) from the spectra obtained.

A 0.1 wt% LLDPE/*o*-di­chloro­benzene solution was prepared for the four types of LLDPE. The polystyrene-equivalent molecular weight and its distribution were determined by gel permeation chromatography (Viscotek Triple Detector HT-GPC Model-SG system, Malvern Instruments Ltd) at 140°C.

### 2.2. Sample preparation

Sheets of 2 mm-thickness were made using a press machine (MINI TEST PRESS-10, TOYOSEIKI). LLDPE pellets were melted in the machine at 180°C and molded into sheets under a pressure of 10 MPa for 10 min. Subsequently, they were quenched by transferring them onto a water-cooled plate at 25°C. The density of the sheets was measured using a helium pycnometer (AccuPyc 1330, Shimadzu) and the crystallinity was calculated from the density. Here, we assumed a two-phase system consisting of a crystalline and an amorphous phase, with densities of 1.00 and 0.855 g cm^−3^, respectively (Brandrup *et al.*, 1999 ▸). The values obtained are listed in Table 1 ▸. For *in situ*X-ray scattering measurements, sandglass-shaped specimens with a center width of 2 mm were punched out from the sheets.

### 2.3. *In situ*X-ray scattering measurements

*In situ*X-ray scattering measurements under tensile tests were performed using an in-house tensile tester with a tensile speed of 1 mm min^−1^. Stress–strain (SS) curves were obtained simultaneously during the *in situ*X-ray scattering experiments. USAXS measurements were performed at the second hutch of beamline BL03XU, SPring-8, Hyogo, Japan (Masunaga *et al.*, 2011 ▸; Kishimoto *et al.*, 2019 ▸). The camera length and the wavelength of the incident beam were 7.5 m and 2.0 Å, respectively, and thus the wavenumber *q*-range of USAXS was 0.0075 < *q* < 0.5 nm^−1^. Here, *q* is the magnitude of the scattering vector **q**, defined by *q* = (4π/λ_w_) sin(θ/2), where λ_w_ and θ are the wavelength of the incident X-rays and the scattering angle, respectively. A Pilatus 1M was used as the detector, and the exposure time for each measurement was 850 ms. Simultaneous SAXS and WAXS measurements were also performed on the same beamline used in the USAXS measurements. The wavelength of the incident X-rays for SAXS and WAXS was 1.0 Å. The Pilatus 1M and a flat panel detector were used for the detectors in the SAXS and WAXS experiments, respectively. The camera lengths for SAXS and WAXS were 2.4 and 120 mm, and 670 and 570 ms, respectively. The *q*-ranges of SAXS and WAXS were 0.06 < *q* < 1.3 nm^−1^ and 4 < *q* < 26 nm^−1^, respectively. The 2D scattering intensities were corrected for air and background scattering. Finally, the intensities were divided by the thickness of the specimens. The thickness during stretching was calculated using the thickness before stretching and the Lambert−Beer law.

## 3. Results and discussion

### 3.1. Hierarchical structures of LLDPE before stretching

We confirmed by NMR that MP-50 and MP-60 contain 4-methyl­pentene-1 (4MP1) and B-50 and B-60 contain butene-1 as a comonomer. MP-50 and B-50 show higher comonomer content and lower crystallinity than MP-60 and B-60, which proves that the crystallinity of the sheets decreases with increasing comonomer content. In addition, MP-50, which has slightly lower density than B-50, contains a lower comonomer content than B-50. This indicates that 4MP1, or the bulkier comonomer, destabilizes the lamellar structures by accumulating near the crystalline surface (Shirayama *et al.*, 1972 ▸; Simanke *et al.*, 2001 ▸).

We analyzed the USAXS, SAXS and WAXS patterns before stretching and obtained the characteristic parameters of the hierarchical structures of the LLDPE samples. All USAXS, SAXS and WAXS patterns were isotropic as shown in Figs. S2, S8 and S13 of the supporting information and Fig. 4 of Kishimoto *et al.* (2020 ▸). There were no peaks in any of the USAXS patterns. The scattering peak originating from the periodicity of lamellar structures was observed in all SAXS patterns. All WAXS patterns exhibited isotropic peaks of the crystal structures of the LLDPE. We calculated the scattering profiles [*I*(*q*)] by averaging the 2D patterns circularly.

**Figure 1:**
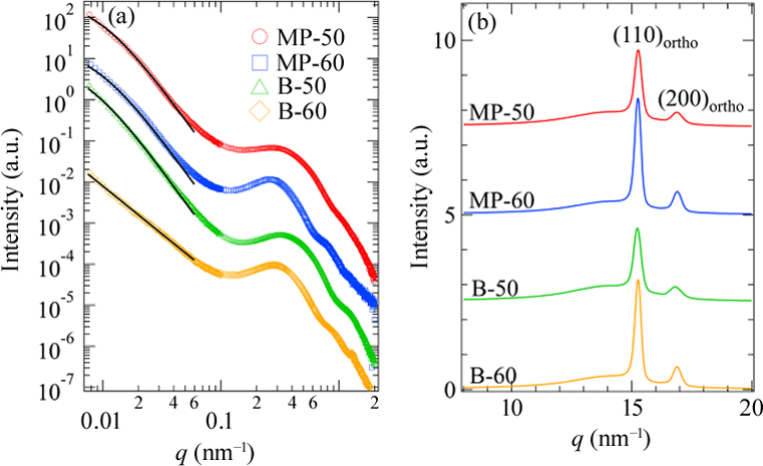
(*a*) Combined USAXS/SAXS profiles and (*b*) WAXS profiles of all samples before the application of strain. The solid lines in part (*a*) indicate the fitted curves. Each profile is shifted vertically for clarity.

Fig. 1 ▸ shows the combined USAXS and SAXS profiles and WAXS profiles for the LLDPE samples. The upturn of the scattered intensity in the USAXS region reflects the density fluctuation on the submicrometre scale. If the samples are filled with grains of the monodisperse lamellar structures, the upturn does not appear in the USAXS region. Thus, the scattering arises from the spatial distribution of the nonuniform lamellar structures. We characterized the amount of density fluctuation on the submicrometre scale by the invariant of the USAXS region (*Q*_U,iso_) defined as
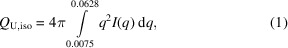
where *Q*_U,iso_ characterizes the nonuniformity of lamellar structures. The calculated *Q*_U,iso_ values are listed in Table 2 ▸. The amount of spatial inhomogeneity on the submicrometre scale decreased with decreasing comonomer content in both comonomer types. The change in the degree of comonomer content for LLDPE with butene-1 or the B-series was greater than that for LLDPE with 4MP1 or the MP-series. B-50 was the most inhomogeneous whereas B-60 was the most homogeneous among all the samples.

We also attempted to estimate the characteristic lengths of the inhomogeneous structures on the submicrometre scale. In the case of MP-60, MP-50 and B-50, *I*(*q*) at *q* < 0.06 nm^−1^ was expressed by the Debye–Bueche function (Debye & Bueche, 1949 ▸)
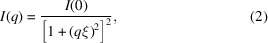
where ξ and *I*(0) are the correlation length of the inhomogeneous structure and *I*(*q*) at *q* = 0, respectively. The values of ξ and *I*(0) are also listed in Table 2 ▸. The values of ξ appear to be insensitive to the comonomer characteristics. After polymerization, comonomers turn into short branches. Since the crystalline phase regards the short branches as impurities, the short branches ejected from the crystalline phase during the crystallization process would induce the inhomogeneous structure. The fact that the USAXS profiles can be fitted with the Debye–Bueche function suggests that the inhomogeneous structure is similar to the phase-separated structure. In the case of B-60, *I*(*q*) at *q* < 0.05 nm^−1^ was expressed by the following power-law behavior:
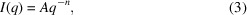
where *A* and *n* are the front factor and mass fractal dimension, respectively. We were not able to find the characteristic length for B-60. The value of *n* for B-60 was 2.3 and the density fluctuation associated with the mass-fractal-like structure existed in the spherulites of B-60 (Takenaka *et al.*, 2007 ▸). The reason B-60 did not have a correlation length is because short-chain branches of butene comonomers are included in the polyethyl­ene crystal structure to some extent (Hosoda *et al.*, 1990 ▸). The small content of comonomer induces few defects in the lamellar structure. Thus, we found relatively homogeneous branched lamellar structures showing mass-fractal behavior in B-60.

In *I*(*q*) at *q* > 0.06 nm^−1^, we found the peaks originating from the lamellar structures around *q* = 0.3 nm^−1^. We estimated the long spacing of the lamellar structures (*d*) after Lorentz correction (Strobl, 2007 ▸). The values obtained are listed in Table 2 ▸. We observed reflections from the (110) and (200) lattice planes in the orthorhombic phase in the WAXS profiles [Fig. 1 ▸(*b*)].

### 3.2. Effects of the comonomer characteristics on strain-induced density fluctuations in LLDPE

In the previous section, we characterized the hierarchical structures of the initial state and found that the inhomogeneous structures on the submicrometre scale varied with comonomer characteristics. In this section, we investigate how the comonomer characteristics affect the SIDF behavior. SS curves for the LLDPE samples are shown in Fig. S1. Here, the vertical axis represents the engineering stress and the horizontal axis represents the draw ratio λ defined as
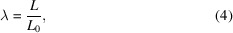
where *L*_0_ and *L* are the initial length of the samples and the length of the samples during applied strain, respectively. Two yield points were observed in all LLDPE samples. We call the yield points at the smaller and larger λ 1YP and 2YP, respectively. Note that necking occurred in all the samples from 2YP and that the scattering results reflected necking.

**Figure 2:**
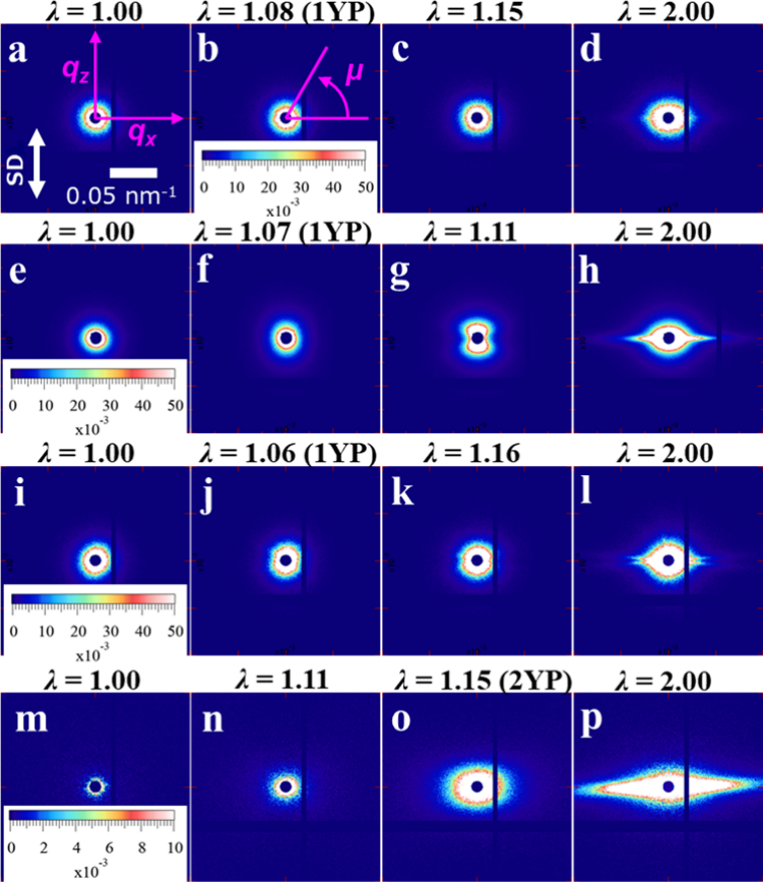
Representative USAXS 2D patterns of (*a*)–(*d*) MP-50, (*e*)–(*h*) MP-60, (*i*)–(*l*) B-50 and (*m*)–(*p*) B-60 with strain. The *q_y_* axis is parallel to the incident beam, that is, normal to these scattering patterns. SD: stretching direction.

Here, we focus on the change in the USAXS patterns. The supporting information presents the changes in hierarchical structures of the LLDPE samples during stretching obtained by the same analytical procedures as in the previous study (Kishimoto *et al.*, 2020 ▸). The USAXS patterns and profiles parallel and perpendicular to the stretching direction of MP-50, MP-60, B-50 and B-60 with strain are shown in Figs. 2 ▸ and 3 ▸. Here, the profiles *I*_||_(*q*) along the parallel direction were obtained by sector averages for 85° < μ < 95° and 265° < μ < 275°, while the profiles *I*_⊥_(*q*) along the perpendicular direction were for −5° < μ < 5° and 175° < μ < 185°. The definition of the azimuthal angle μ is shown in Fig. 2 ▸(*b*).

First, we briefly describe the structural changes observed in MP-60 by USAXS as reported in our previous study (Kishimoto *et al.*, 2020 ▸). MP-60 exhibited the abnormal butterfly pattern that characterizes the SIDF after 1YP as shown in Fig. 2 ▸(*g*). The appearance of the abnormal butterfly pattern originates from the inhomogeneous deformation as observed in other systems (Shinohara *et al.*, 2007 ▸; Takenaka, 2013 ▸; Hashimoto *et al.*, 2019 ▸). In the case of polyethyl­ene, this pattern reflects the difference in the amount of deformation between the high-crystallinity and low-crystallinity regions on the scale of the lamellar branching structure. The density fluctuations can exceed the density difference between crystalline and amorphous phases, which suggests that strain reduces the density of the amorphous phase from its initial value. The butterfly pattern finally transformed into a streak pattern after 2YP as shown in Fig. 2 ▸(*h*), indicating that the low-crystallinity region was transformed into voids elongated along the parallel direction at 2YP.

**Figure 3:**
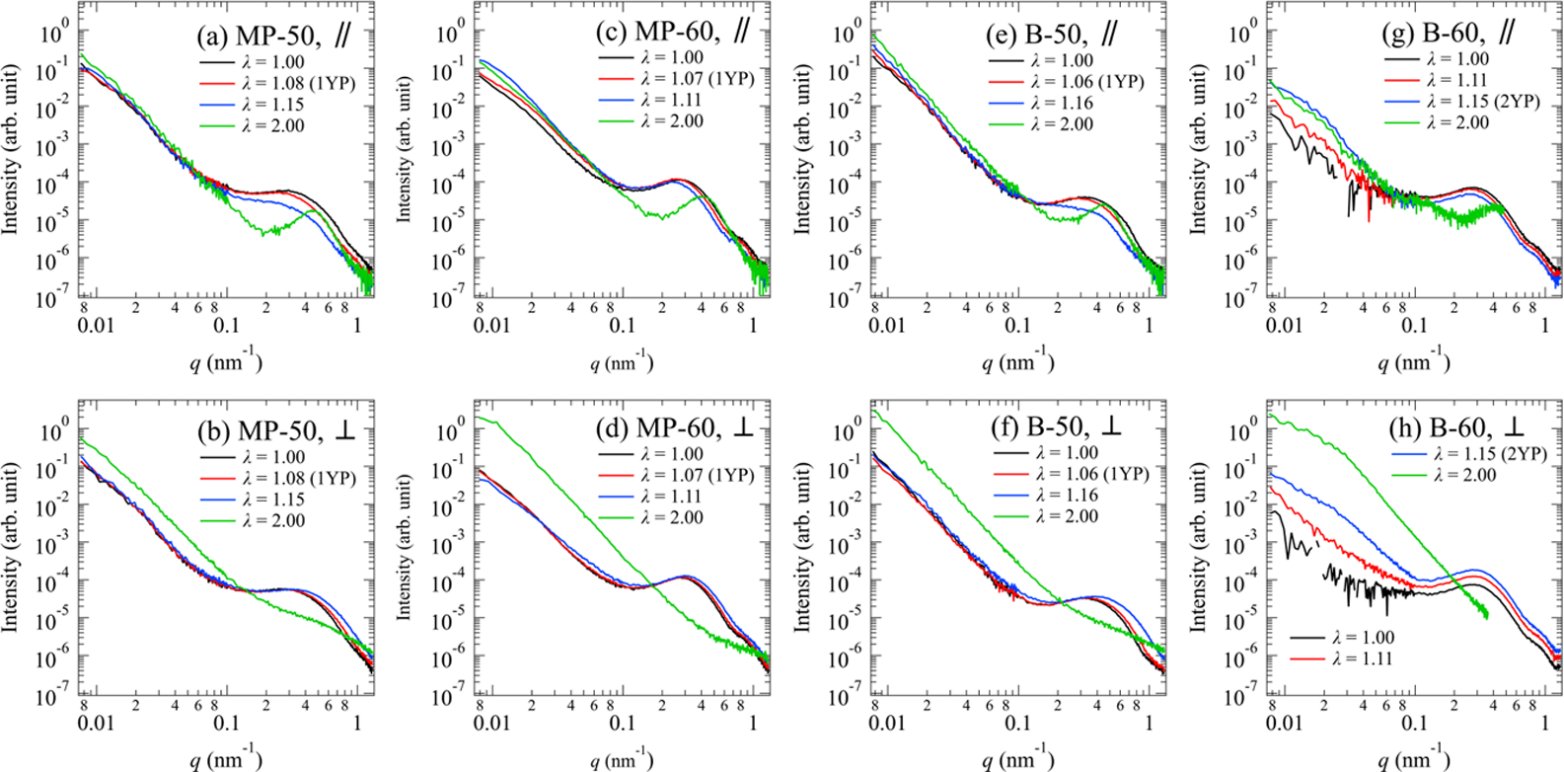
Combined USAXS and SAXS profiles for (*a*) and (*b*) MP-50, (*c*) and (*d*) MP-60, (*e*) and (*f*) B-50, and (*g*) and (*h*) B-60. The profiles in (*a*), (*c*), (*e*) and (*g*) and those in (*b*), (*d*), (*f*) and (*h*) correspond to the scattering intensities parallel and perpendicular to the stretching direction, respectively.

In contrast to MP-60, MP-50 did not exhibit the butterfly pattern as shown in Figs. 2 ▸(*a*)–2 ▸(*c*). The profiles in Figs. 3 ▸(*a*) and 3 ▸(*b*) also indicate that changes in the submicrometre structure did not occur with strain. In the large-strain region, we obtained very weak streak scattering as seen in Fig. 2 ▸(*d*), suggesting that a much smaller number or size of voids could be generated in MP-50 than in MP-60.

In the case of B-50, we observed the butterfly pattern with strain in Figs. 2 ▸(*j*) and 2 ▸(*k*). *I*_||_(*q*) in Fig. 3 ▸(*e*) also increased with strain. Finally, the streak pattern [Fig. 2 ▸(*l*)] and the corresponding enhancement in *I*_⊥_(*q*) [Fig. 3 ▸(*f*)] were observed. These results support the emergence of voids associated with the SIDF.

In the case of B-60, the butterfly pattern did not appear, but an elliptical pattern with a major axis perpendicular to the stretching direction developed until 2YP in Figs. 2 ▸(*m*)–2 ▸(*o*). After 2YP, strong streak scattering appeared along the direction perpendicular to the stretching direction [Fig. 2 ▸(*p*)]. These results suggest that the SIDF did not occur with strain. Instead, the initial density fluctuations on the submicrometre scale were elongated in the stretching direction and the elongated fluctuations became voids.

**Figure 4:**
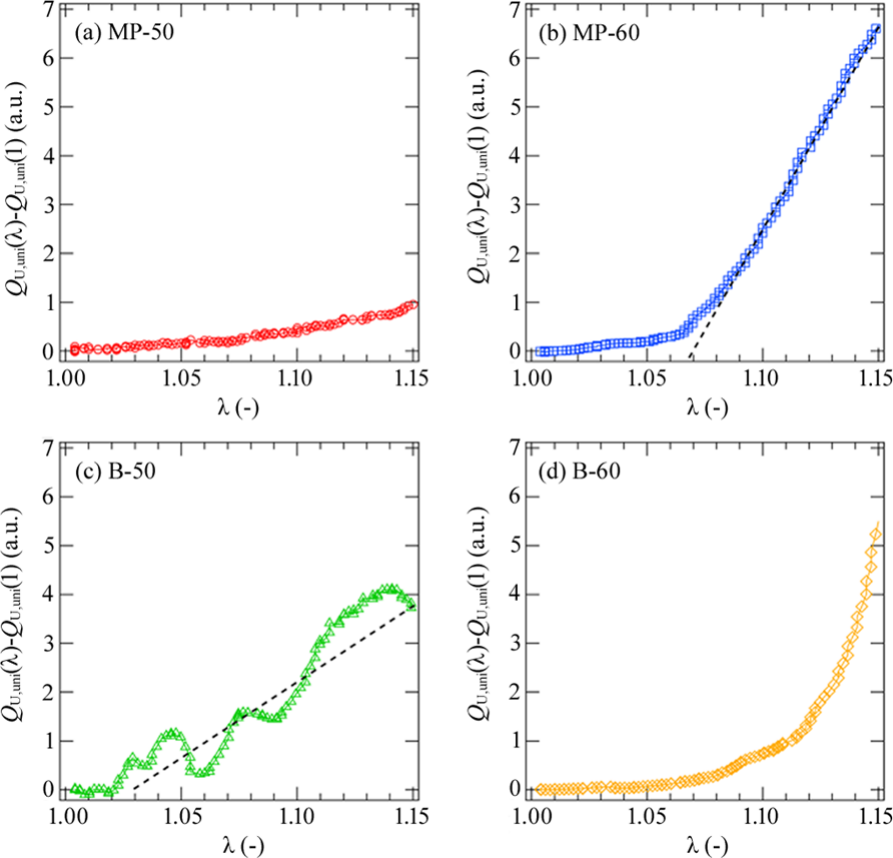
Evolution of the SIDF characterized by the invariant during uniaxial stretching in (*a*) MP-50, (*b*) MP-60, (*c*) B-50 and (*d*) B-60. The broken lines are guides to the eyes.

To analyze the behaviors of the SIDF quantitatively, we calculated the changes in the invariant during stretching. We assume the scattering intensity distribution is symmetrical along the *q_z_* axis or the stretching axis under uniaxial stretching and calculated the invariant on the submicrometre scale during uniaxial stretching (*Q*_U,uni_) from the USAXS patterns using
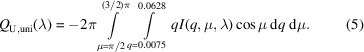
Fig. 4 ▸ shows the changes in *Q*_U,uni_(λ) with strain. A drastic increase in *Q*_U,uni_(λ) was found in MP-60 and B-50 exhibiting the butterfly patterns, while *Q*_U,uni_(λ) became almost constant with strain at λ < 1.15 for MP-50. An upturn in *Q*_U,uni_(λ) in association with the butterfly patterns was seen approximately from λ = 1.03 in B-50 and from λ = 1.06 in MP-60. The growth rates are indicated by broken lines in Fig. 4 ▸. The growth rate of MP-60 was larger than that in B-50, suggesting the SIDF of MP-60 was more distinct than that of B-50. As for the case of B-60, a drastic increase in *Q*_U,uni_(λ) was observed. However, this increase was caused by the formation of voids and the origin of the increase was not the SIDF as described before. Note that the oscillation observed in *Q*_U,uni_(λ) of B-50 is derived from the experimental errors in the ion chamber employed for tracking the sample thickness and not from the microstructure of the samples.

As shown in Fig. 4 ▸, the SIDF occurred in MP-60 and B-50, whereas MP-50 did not show SIDF behaviors. The SIDF in MP-60 was stronger than that in B-50. As described previously, the driving force is proportional to the gradient of the stress fields and increases with the inhomogeneity or the invariant at the initial state. Thus, the absence of the SIDF in B-60 is attributed to the homogeneous submicrometre structure at the initial state. However, we cannot explain the following facts: (i) MP-50 did not show the SIDF though the invariant at the initial state is large, (ii) the SIDF of MP-60 is stronger than that of B-50 even though the invariant at the initial state is small. The facts indicate that the stress fields changed during stretching. The stress fields depend on the crystalline structures in crystalline polymers. The destruction of crystalline structures associated with strain might induce homogenization of the stress field and cause the suppression of the SIDF. A decrease in the crystallinity with strain or mechanical melting was first proposed by Flory & Yoon (1978 ▸). Since then, mechanical melting has been discussed not only in polyethyl­ene but also in other polymers (Lucas *et al.*, 1995 ▸; Butler *et al.*, 1997 ▸; Rosa *et al.*, 2007 ▸). We investigated the behaviors of the mechanical melting during stretching to clarify whether the homogenization occurs with the destruction.

### 3.3. Mechanical melting during stretching

To analyze the mechanical melting behavior quantitatively, we calculated the changes in the crystallinity with strain from the WAXS data. The details of the estimation of the crystallinity of LLDPE during uniaxial stretching (*X*_c,uni_) are described in our previous paper (Arakawa *et al.*, 2022 ▸).

**Figure 5:**
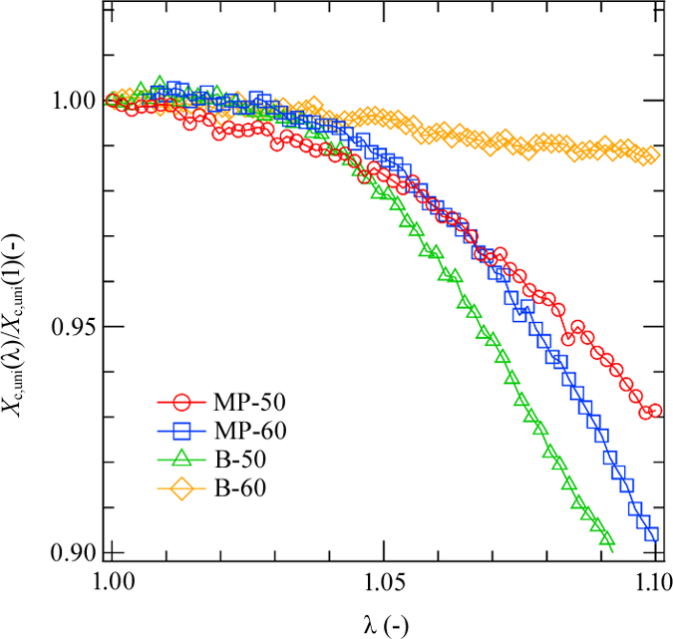
λ dependence of *X*_c,uni_(λ)/*X*_c,uni_(1) of all samples.

Fig. 5 ▸ shows the reduced crystallinity *X*_c,uni_(λ)/*X*_c,uni_(1) plotted as a function of strain. *X*_c,uni_(λ)/*X*_c,uni_(1) of MP-50 started decreasing after the onset of stretching, reflecting that the mechanical melting started immediately on the application of strain. The mechanical melting reduced the fluctuations of the stress fields in the sample so that the SIDF did not occur in MP-50. On the other hand, the crystallinity of B-60 remained almost constant with strain and the lamellar structures changed slightly with strain at λ < 1.1 as shown in Fig. S15(*a*). These results indicate that the increase in USAXS intensity was caused by the formation of the void originating from the defect.

In the case of MP-60 and B-50, the crystallinity remained almost constant in the early stage of the stretching process or λ < 1.05. Thus, the SIDF occurred since the reduction of the fluctuations of the stress fields was not induced by the mechanical melting. However, in the later stage or when λ > 1.05, the mechanical melting occurred in both MP-60 and B-50. The decrease in the crystallinity of B-50 is more pronounced than that of MP-60. Thus, the SIDF in MP-60 was stronger than that in B-50. These melting behaviors depended on the species and content of the comonomer. A bulky comonomer and a large comonomer content induce melting during stretching.

## 4. Conclusions

To clarify the factors governing the SIDF in semicrystalline materials, we investigated the changes in the submicrometre structures of LLDPE with different comonomer species and content during uniaxial stretching by time-resolved USAXS as well as changes in the lamellar structures and lattice structures by time-resolved SAXS and WAXS.

The changes in the hierarchical structures in all samples were classified into the following three cases: (i) the case in which the sample had a homogeneous structure on the submicrometre scale before stretching (*i.e.* B-60), (ii) the case in which the sample had an inhomogeneous submicrometre structure at the initial state but the crystalline structure melted with strain at the early stage of stretching (*i.e.* MP-50), and (iii) the case in which the sample had an inhomogeneous submicrometre structure at the initial state and the mechanical melting started at the later stage of stretching (*i.e.* MP-60 and B-50). In the case of (i) and (ii), the butterfly pattern that characterizes the occurrence of the SIDF was not observed. In the case of (iii), the sample exhibited the butterfly pattern and the intensity was governed by the degree of mechanical melting. We can conclude that samples with distinct characteristic lengths on the submicrometre scale at the initial state cause SIDF unless mechanical melting occurs. On the other hand, the long spacing of the lamellar structures at the initial state does not correlate with SIDF and mechanical melting. The degree of mechanical melting depends on the species and content of the comonomer. The inhomogeneity of the submicrometre structure at the initial state would depend not only on the comonomer characteristics but also on the crystallization conditions.

Note that all tensile tests in this study were conducted under a given strain rate. Furukawa & Tanaka (2009 ▸) suggested that inhomogeneous flow occurs above a critical strain rate. In fact, it is known that the strain rate affects the generation of voids and necking in polyethyl­ene (Strobl, 2007 ▸; Kuriyagawa & Nitta, 2011 ▸). In future work we need to investigate the effects of strain rates on the SIDF.

## Supplementary Material

Supporting information file. DOI: 10.1107/S1600576725002183/jl5105sup1.pdf

## Figures and Tables

**Table 1:** Characterization of materials

Name	Comonomer species	Comonomer content (mol%)	*M*_w_	*M*_w_/*M*_n_	Density of the sheet at 20°C (g cm^−3^)	Crystallinity (wt%)
MP-50	4-Methyl­pentene-1	3.00	2.0 × 10^5^	4.2	0.919	48
MP-60	4-Methyl­pentene-1	0.80	2.1 × 10^5^	5.3	0.937	60
B-50	Butene-1	3.46	2.2 × 10^5^	3.2	0.920	49
B-60	Butene-1	1.93	2.2 × 10^5^	4.7	0.933	58

**Table 2:** Evaluated parameters

Name	*Q*_U,iso_ (a.u.)	*I*(0) (a.u.)	ξ (nm)	*d* (nm)
MP-50	1.62 × 10^−6^	0.295	109	15.2
MP-60	9.92 × 10^−7^	0.199	112	19.8
B-50	1.94 × 10^−6^	0.884	144	13.7
B-60	3.86 × 10^−7^	–	–	17.8

## Data Availability

The authors confirm that the data supporting the findings of this study are available within the article and/or its supporting information.
