# Supplementary material for: Strain-induced density fluctuations in linear low-density polyethyl­ene
Source: J Appl Crystallogr. 2025 Apr 4;58(Pt 3):879–85. doi: 10.1107/S1600576725002183 (PMC12135982; doi:10.1107/S1600576725002183)
Supplement: Supplementary file 1 [file j-58-00879-sup1.pdf]

JOURNAL OF
APPLIED
CRYSTALLOGRAPHY

Volume 58 (2025)

Supporting information for article:

Strain-Induced Density Fluctuations in Linear Low-Density Polyethylene

Mizuki Kishimoto, Kazuki Mita, Hiroki Ogawa, Kazuki Shibasaki, Masato Arakawa and Mikihito Takenaka

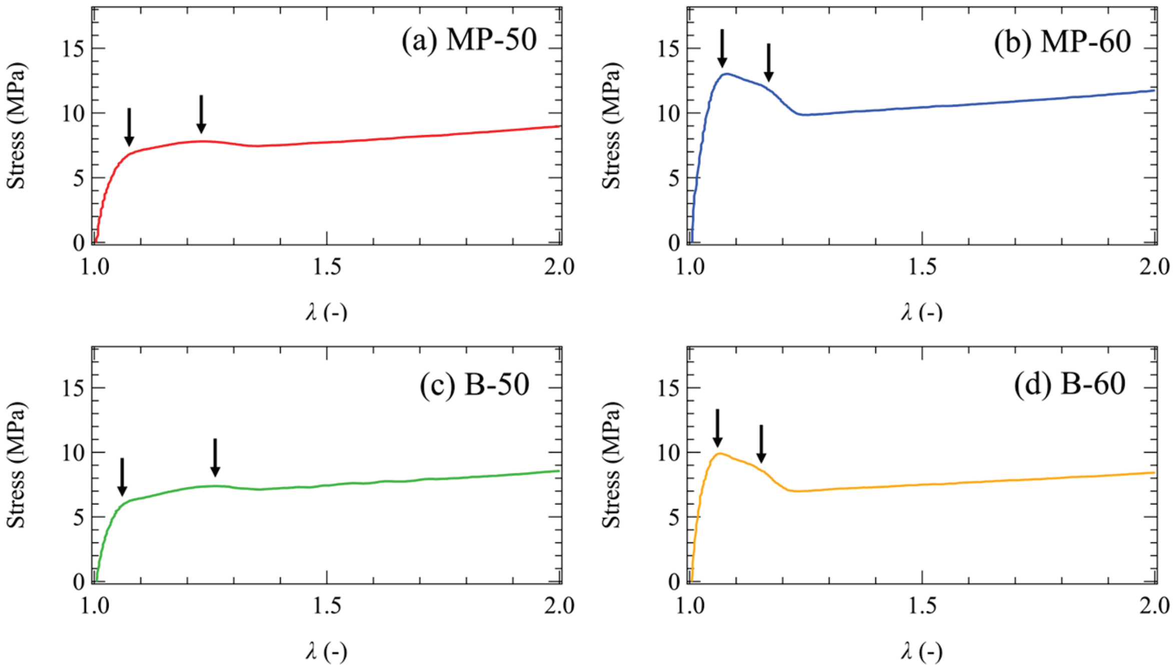

Figure S1 S-S curves of all samples. The arrows indicate the yield points.

Changes in the hierarchical structure of MP-50

Figure S2 shows the changes in the 2D USAXS, SAXS, and WAXS patterns as a function of the strain for MP-50. We also show the combined USAXS and SAXS profiles along with the directions parallel and perpendicular to the stretching axis for MP-50 in Figure S3. We estimated the long spacings along the parallel ($D_{\parallel}$) and perpendicular ($D_{\perp}$) directions by using the following equation:

$$D_i = \frac{2\pi}{q_{\mathrm{m},i}} \quad i = \parallel, \perp \qquad (6)$$

where $q_{\mathrm{m},\parallel}$ and $q_{\mathrm{m},\perp}$ are the peak positions. We obtained $q_{\mathrm{m},\parallel}$ and $q_{\mathrm{m},\perp}$ by fitting $I_{\parallel}(q)$ and $I_{\perp}(q)$, respectively, to a linear combination of a power law and Gaussian function.

The changes in the scattering patterns with strain can be classified into the following 4 regions. Region I: strain domain before the 1YP or at $\lambda < 1.08$, Region II: strain domain between the 1YP and 2YP or at $1.08 < \lambda < 1.23$, Region III: strain domain after the 2YP or at $1.23 < \lambda < 1.30$, and Region IV: strain domain at $1.30 < \lambda$.

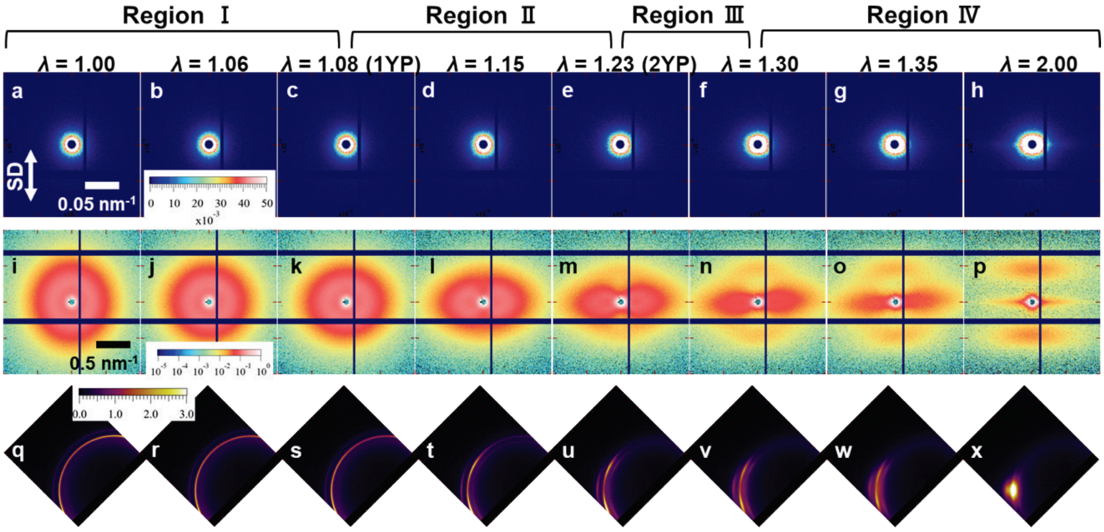

Figure S2 (a)-(h) USAXS, (i)-(p)SAXS, and (q)-(x)WAXS 2D patterns of MP-50 with strain. The arrow in part(a) corresponds to the stretching direction.

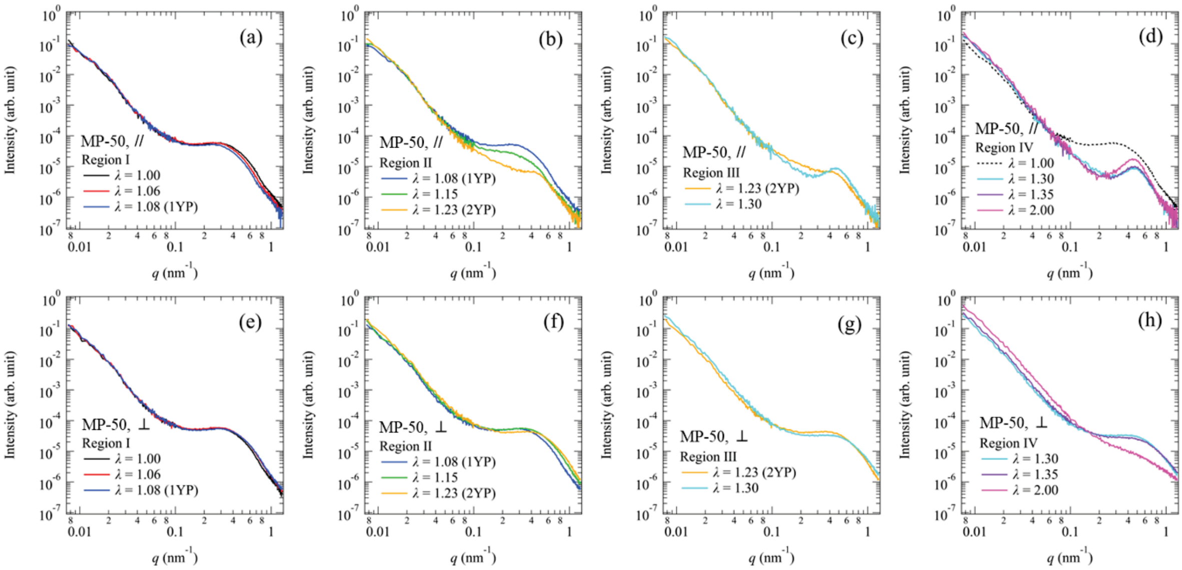

Figure S3 Combined USAXS and SAXS profiles from MP-50 in (a) and (e) Region I, (b) and (f) Region II, (c) and (g) Region III, and (d) and (h) Region IV, respectively. The profiles in (a), (b), (c), and (d) and (e), (f), (g), and (h) correspond to the scattering intensities parallel and perpendicular to the stretching directions, respectively.

Region I

In Region I ($\lambda < 1.08$), the strain induced the deformation and mechanical melting of the lamellar structures. The USAXS patterns of the LLDPE (Figures S2(a)-(c)) hardly changed with strain. Both $I_{||}(q)$ (Figure S3(a)) and $I_{\perp}(q)$ (Figure S3(e)) at $q < 0.05$ nm^{-1} did not change with strain. The changes in the profiles in the small q-region indicate that the submicron structure was not deformed in this region. Contrary to the results of USAXS, the SAXS pattern (Figures S2(i)-(k)) was slightly elongated

to the direction perpendicular to the stretching direction. The peak positions of $I_{\parallel}(q)$ and $I_{\perp}(q)$ shifted toward smaller q and larger q, respectively, with the strain. We plotted the ratios $D_i(\lambda)/D_i(1)$ as a function λ in Figure S4(a). The solid and dashed lines obtained by the following equations assuming affine deformation:[15]

$$\frac{D_{\parallel}(\varepsilon)}{D_{\parallel}(1)} = \lambda \quad (7)$$

$$\frac{D_{\perp}(\varepsilon)}{D_{\perp}(1)} = \lambda^{-\frac{1}{2}} \quad (8)$$

The amount of the deformation parallel to the stretching direction is larger than that estimated under the assumption of affine deformation. Mechanical melting enhanced the increase in the long spacing. Figure S4(b) shows the μ dependence of the peak intensities of the SAXS patterns. We fitted sector averages at every 10˚ to a linear combination of a power law and Gaussian function and plotted the obtained front factors of the Gaussian function against μ. Figure S4(b) shows that overall intensity decreased with strain. The result indicates that mechanical melting started in this region. Figure S4(c) shows the μ dependence of the WAXS intensity from $(110)_{\text{ortho}}$ reflection. The reflection at $\mu = 90$ ° decreased with strain, suggesting that the crystalline structure stacked along the perpendicular direction was more favorable for mechanical melting than that stacked along the parallel direction. Figure S4(b) shows that two peaks appeared at $0 < \mu < 180°$ around the 1YP, indicating the formation of diagonal pattern, or so-called four-point pattern. The patterns attributed to the chevron-type morphology induced by the fragmentation and rotation of the lamellar structures. As shown in Figure S4(c), The μ dependence of the WAXS intensity from $(110)_{\text{ortho}}$ reflection also split around the 1YP. The changes in SAXS and WAXS patterns indicate lamellar fragmentation and rotation started around the 1YP. After the 1YP, the small peak from (010) plane in the monoclinic phase appeared along the parallel direction (Figure S5). This transformation was also observed in MP-60. The fine slip on the $(110)_{\text{ortho}}$ plane causes the transformation into the monoclinic phase.

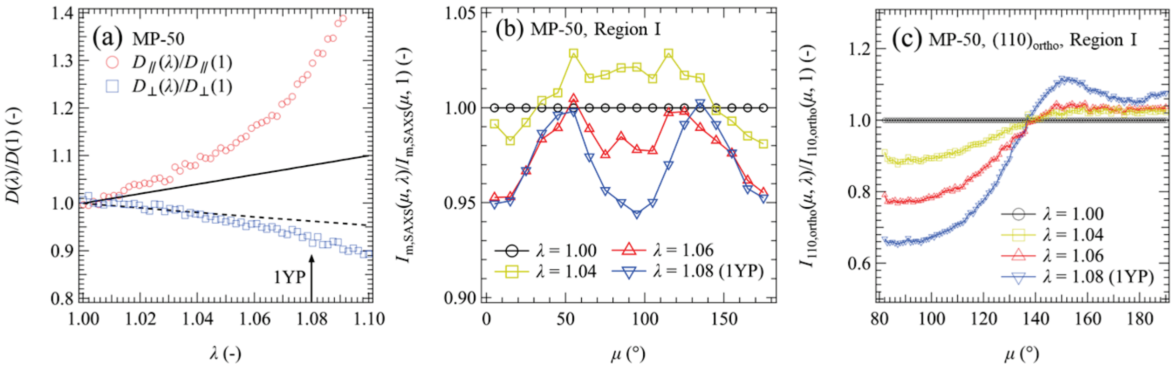

Figure S4 (a) λ dependence of $D_{//}(\lambda)/D_{//}(1)$ and $D_{\perp}(\lambda)/D_{\perp}(1)$ of MP-50. The solid and dashed lines are the lines calculated by Eqs. (7) and (8) obeying affine deformation. (b) μ dependence of the reduced peak intensity $I_{m,SAXS}(\mu, \lambda)/I_{m,SAXS}(\mu, 1)$ of SAXS patterns and (c) $I_{110,ortho}(\mu, \lambda)/I_{110,ortho}(\mu, 1)$ in WAXS patterns in Region I in MP-50.

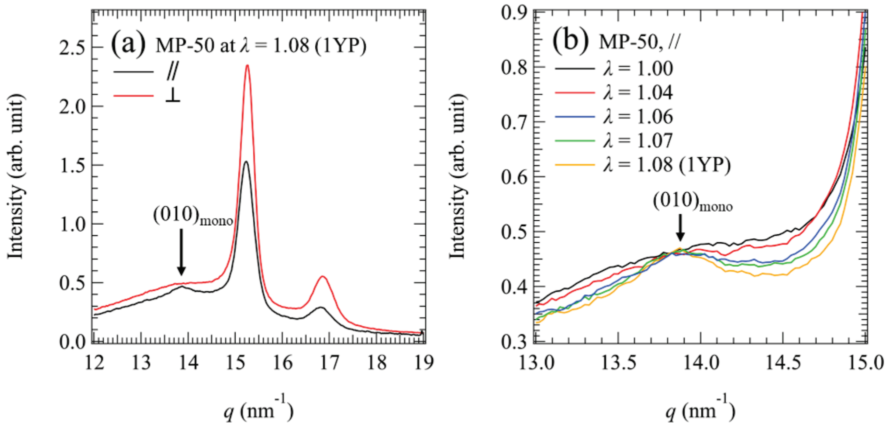

Figure S5 (a) WAXS profiles parallel and perpendicular to the stretching directions at λ = 1.08 and (b) evolution of the diffraction intensities from the (010) plane in the monoclinic crystals.

Region II

In Region II (1.08 < λ < 1.23), the inhomogeneous structure on the submicron scale hardly changed with strain, and a chevron-type morphology was developed. In the USAXS patterns (Figures S2(c)-(e)), we hardly saw the change. In the profiles of USAXS region, we found the shift of the profiles to smaller q-region, indicating that the elongation of the inhomogeneous structure along the parallel direction might occur with strain. In SAXS patterns (Figures S2(k)-(m)), we clearly observed the four-point patterns. Reflecting the rotation and orientation in the diagonal direction of lamellar structures, the $(110)_{ortho}$ peak in the WAXS patterns (Figures S2(s)-(u)) was oriented in the diagonal direction. The μ dependence (Figure S6(a)) clearly shows the change in the orientation. We also found the development of the $(200)_{ortho}$ peak in the perpendicular directions (Figure S6(b)). The perpendicular orientation of $(200)_{ortho}$ planes and the diagonal orientation of the $(110)_{ortho}$ planes indicate that fragmentation occurred on the $(110)_{ortho}$ planes. Moreover, the $(010)_{mono}$ planes oriented

along with the $(110)_{ortho}$ plane (Figure S6(c)). Thus, the WAXS patterns indicate that both lamellar fragmentation (coarse slip) and fine slip occurred on the $(110)_{ortho}$ plane.

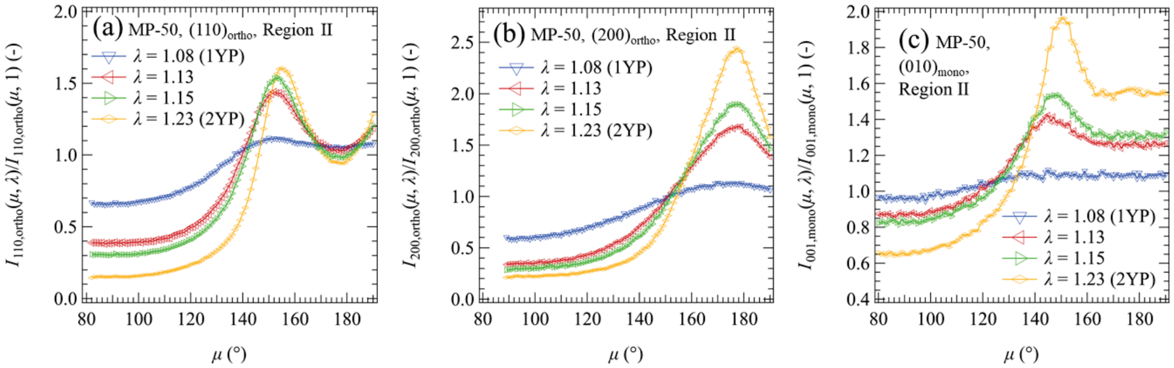

Figure S6 μ dependence of (a) $I_{110,ortho}(\mu, \lambda)/I_{110,ortho}(\mu, 1)$, (b) $I_{200,ortho}(\mu, \lambda)/I_{200,ortho}(\mu, 1)$, and (c) $I_{010,mono}(\mu, \lambda)/ I_{010,mono}(\mu, 1)$ in WAXS patterns in Region II in MP-50.

Region III

In Region III, the inhomogeneous structure on the submicron scale elongated along the parallel direction, and the chevron-type morphology transformed into a fibrillar structure. The fibrillar structure included the recrystallized lamellar structure aligned in the parallel direction. The long spacing of the recrystallized lamellar structure was shorter than the original lamellar structures. As shown in Figures S2(f)-(h), the USAXS intensity elongated to the perpendicular direction. Thus, the inhomogeneous structures elongated to the direction parallel to the stretching direction. In the SAXS patterns (Figures S2(n)-(p)), we found the decrease in the four-point pattern with strain, associating with the crystalline phase melted mechanically. At approximately $\lambda = 1.30$, a broad peak appeared at approximately $q = 0.4$ nm^{-1} in the parallel direction. This peak reflects the recrystallized lamellar structure stacked with short spacings along the parallel direction. The WAXS patterns (Figures S2(v)-(x)) and μ dependences of the diffraction peaks (Figure S7(a)) show the $(110)_{ortho}$ peak corresponding to the four-point pattern decreased while the peak intensity at μ =180° increased with strain, indicating the formation of a fibrillar structure.

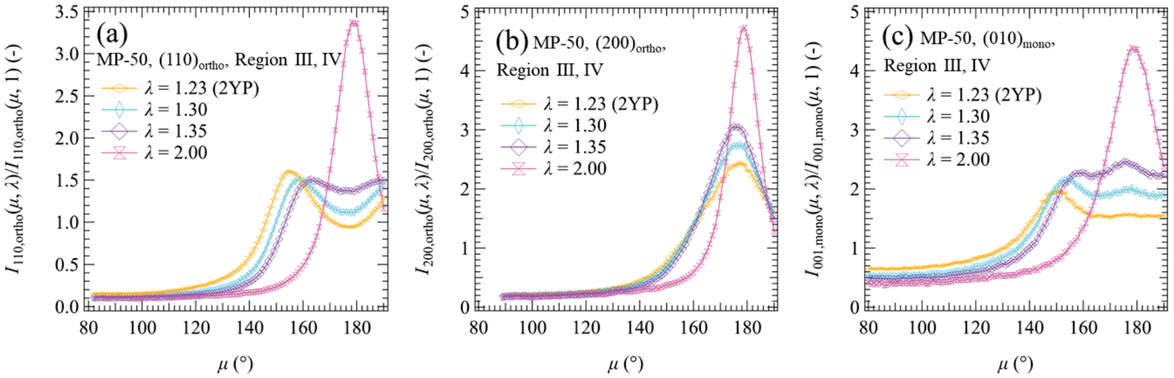

Figure S7 μ dependence of (a) $I_{110,ortho}(\mu, \lambda)/I_{110,ortho}(\mu, 1)$, (b) $I_{200,ortho}(\mu, \lambda)/I_{200,ortho}(\mu, 1)$, and (c) $I_{010,mono}(\mu, \lambda)/I_{010,mono}(\mu, 1)$ in WAXS patterns in Region III and IV in MP-50.

Region IV

In Region IV, we observed the streak in the USAXS pattern (Figure S2(h)), indicating that the elongated voids appeared. The chevron-type morphology transformed into a fibrillar structure completely, which was shown in the disappearance of the four-point pattern in the SAXS pattern (Figure S2(p)). The peaks at μ =180° in the WAXS patterns increased with the strain, as shown in Figure S7, suggesting that the orientation of the lattice structure along the parallel direction progressed with the strain.

Changes in the hierarchical structure of MP-60

See the results of the LLDPE in Kishimoto *et al.*, 2020.

Changes in the hierarchical structure of B-50

The 2D USAXS, SAXS, and WAXS patterns are shown in Figures S8 as a function of the strain for B-50. The combined USAXS and SAXS profiles along with the directions parallel and perpendicular to the stretching axis for the B-50 are also shown in Figures S9. The changes in the scattering patterns with strain can be classified into the following 3 regions. Region I: strain domain at $\lambda < 1.04$ ($\lambda = 1.04$ is not at 1YP), Region II: strain domain at $1.04 < \lambda < 1.26$ (2YP), Region III: strain domain after the 2YP or at $1.26 < \lambda$.

Region I

In Region I (λ < 1.04), the strain induced the inhomogeneous deformation on the submicron scale as well as the deformation of the lamellar structures. In the USAXS patterns (Figures S8(a)-(c)), the intensity along the parallel direction increased with strain. The increase along strain direction indicates the inhomogeneous deformation between the high-crystallinity and low-crystallinity region on the submicron scale, but a clear butterfly pattern was not observed. The plot of $D_i(\lambda)/D_i(1)$ vs λ in Figure S10(a) deviated from affine deformation even smaller λ in Region I as in the case of MP-50. On the other hand, mechanical melting did not occur in B-50 in contrast to in MP-50 (see Figure S10(b)). The reason for the deviation from affine deformation was the inhomogeneous deformation. The lamellar structures in the low-crystallinity region deformed larger than that in the high-crystallinity region, and the deviation reflected the lamellar structures in the low-crystallinity region.

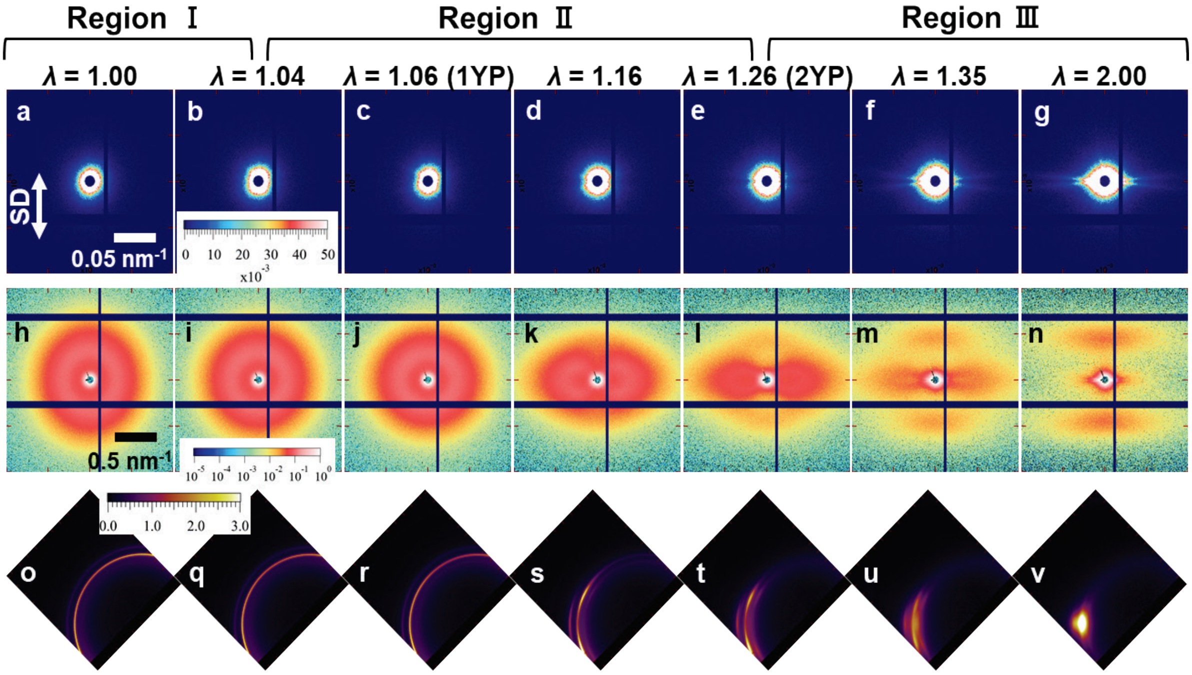

Figure S8 (a)-(h) USAXS, (i)-(p)SAXS, and (q)-(x)WAXS 2D patterns of B-50 with strain. The arrow in part(a) corresponds to the stretching direction.

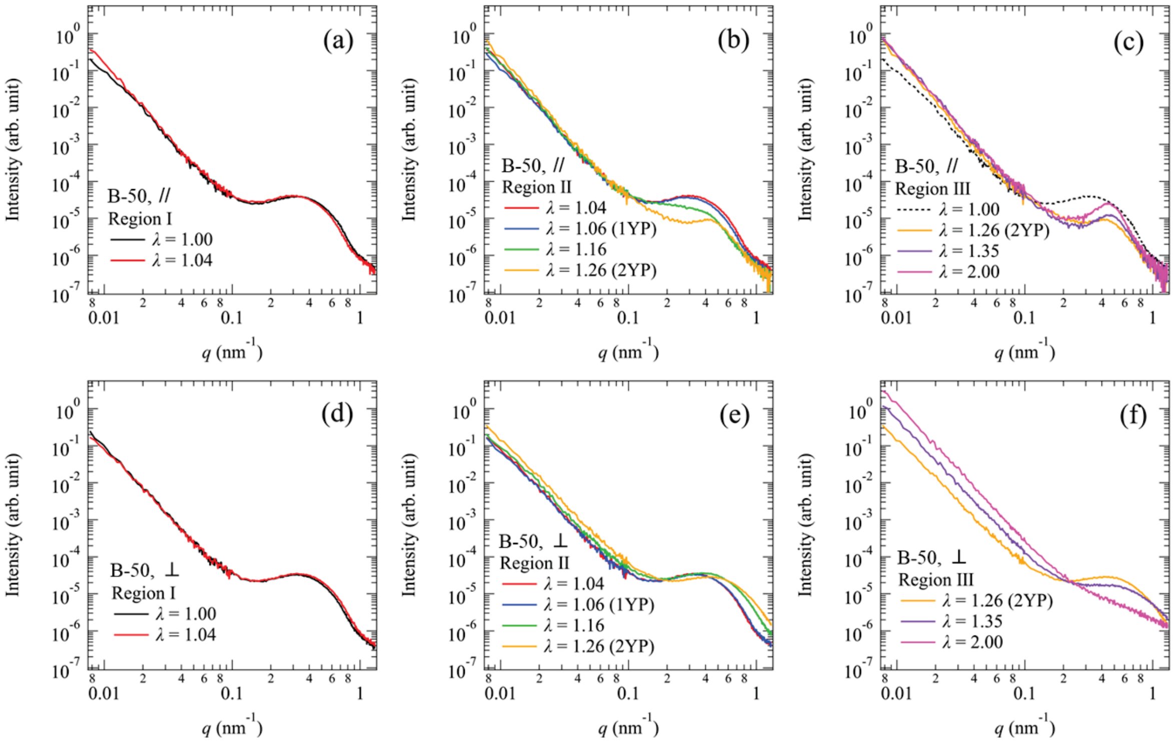

Figure S9 Combined USAXS and SAXS profiles from B-50 in (a) and (d) Region I, (b) and (e) Region II, and (c) and (f) Region III, respectively. The profiles in (a), (b), and (c) and (d), (e), and (f) correspond to the scattering intensities parallel and perpendicular to the stretching directions, respectively.

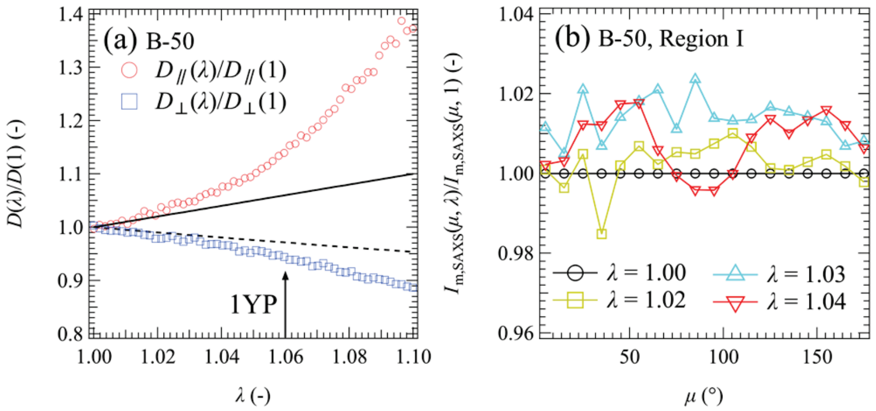

Figure S10 (a) λ dependence of $D_{//}(\lambda)/D_{//}(1)$ and $D_{\perp}(\lambda)/D_{\perp}(1)$ of B-50. The solid and dashed lines are the lines calculated by Eqs. (7) and (8) obeying affine deformation. (b) μ dependence of the reduced peak intensity $I_{m,SAXS}(\mu, \lambda)/I_{m,SAXS}(\mu, 1)$ of SAXS patterns in Region I in B-50.

Region II

In Region II ($1.04 < \lambda < 1.26$ (2YP)), the strain developed a chevron-type morphology and enhanced the density fluctuations on the submicron scale. We observed the abnormal butterfly patterns in USAXS patterns and profiles (Figures S8(c)-(e) and S9(b)), originating from the

inhomogeneous deformation as described above. In the SAXS pattern (Figures S8(j)-(l)) we observed the development of four-point patterns characterizing the lamellar fragmentation and rotation. Besides, the azimuthal dependence of the SAXS peak in Figure S10(a) indicates that mechanical melting occurred in Region II. The WAXS peaks (Figures 8(r)-(t)) were also split and the $(110)_{ortho}$ and $(010)_{mono}$ planes oriented diagonally (Figures S11(b) and (d)) according to the development of the chevron-type morphology. In contrast, the orientation of the $(200)_{ortho}$ plane in μ = 180˚ progressed (Figure S11(c)) as in MP-50, reflecting the realignment of the polymer chains in the mechanically melted region.

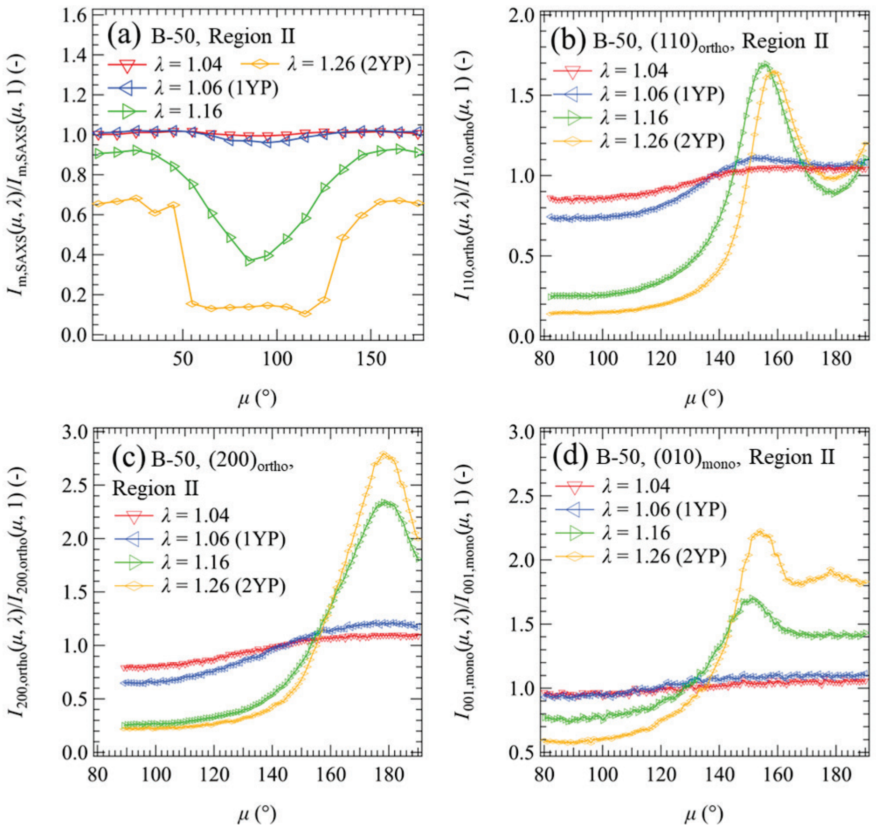

Figure S11 μ dependence of (a) the reduced peak intensity $I_{m,SAXS}(\mu, \lambda)/I_{m,SAXS}(\mu, 1)$ of SAXS patterns, (b) $I_{110,ortho}(\mu, \lambda)/I_{110,ortho}(\mu, 1)$, (c) $I_{200,ortho}(\mu, \lambda)/I_{200,ortho}(\mu, 1)$, and (d) $I_{010,mono}(\mu, \lambda)/I_{010,mono}(\mu, 1)$ in WAXS patterns in Region II in B-50.

Region III

In Region III, inhomogeneous structure or voids in submicron scale elongated along the parallel direction, and the chevron-type morphology transformed into the fibrillar structure. We found the streaks elongated to the perpendicular direction in USAXS patterns as shown in Figures S8(g)-(i), and profiles in Figure S9(f). The change in the USAXS pattern indicates the inhomogeneous structures or

voids elongated to the direction parallel to the stretching direction. In the SAXS patterns (Figures S8(l)-(n)), we found the decrease in the four-point pattern with strain, associating with the crystals melted mechanically. At the 2YP, a broad peak increased at approximately q = 0.4 nm^{-1} in the parallel direction with strain, supporting the formation of the fibrillar structure. The WAXS patterns (Figures S8(t)-(v)) and μ dependences of the diffraction peaks (Figures S12(a) and (c)) show the $(110)_{ortho}$ and $(010)_{mono}$ peaks corresponding to the four-point pattern decreased while the peak intensity at μ = 180 ° increased with strain. The μ dependence of the $(200)_{ortho}$ peak (Figure S12(b)) was not split and the intensity at μ = 180 ° monotonically increased with strain. These changes in the WAXS patterns also supported the formation of a fibrillar structure.

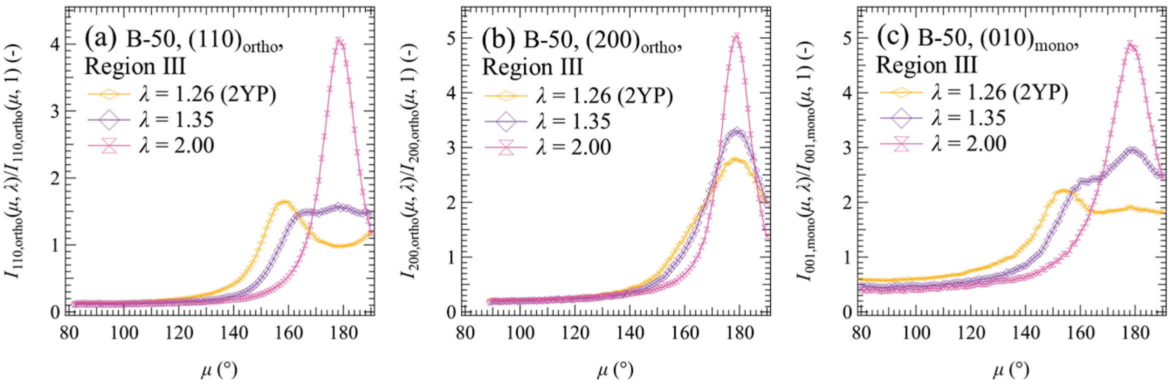

Figure S12 μ dependence of (a) $I_{110,ortho}(\mu, \lambda)/I_{110,ortho}(\mu, 1)$, (b) $I_{200,ortho}(\mu, \lambda)/I_{200,ortho}(\mu, 1)$, and (c) $I_{010,mono}(\mu, \lambda)/I_{010,mono}(\mu, 1)$ in WAXS patterns in Region III in B-50.

Changes in the hierarchical structure of B-60

The 2D USAXS, SAXS, and WAXS patterns are shown in Figures S13 as a function of the strain for B-50. The combined USAXS and SAXS profiles along with the directions parallel and perpendicular to the stretching axis for the B-50 are also shown in Figures S14. The changes in the scattering patterns with strain can be classified into the following 4 regions. Region I: strain domain before the 1YP or at λ < 1.07 (1YP), Region II: strain domain at 1.07 (1YP) < λ < 1.15 (2YP), Region III: strain domain after the 2YP or at 1.15 < λ <1.40, and Region IV: strain domain at 1.40 < λ.

Region I

In Region I (λ < 1.07 (1YP)), strain induced affine deformation in lamellar structures and elongation of the inhomogeneous structures on the submicron scale. Little change was observed in USAXS (Figures S13(a) and (b)) and SAXS patterns (Figures S13(i) and (j)) with strain in Region I.

However, we were able to observe the changes in $I_{\|}(q)$ and $I_{\perp}(q)$ (Figures S14s(a) and (e)). $I_{\perp}(q)$ in the USAXS region increased with strain while $I_{\|}(q)$ in the USAXS region did not change, suggesting that the affine deformation occurred on the submicron scale. As shown in Figure S15(a), the changes in $D_{\|}$ and $D_{\perp}$ showed the affine deformation in the SAXS region as well. The μ dependence of the SAXS peak intensities (Figure S15(b)) did not show the four-point pattern while it had a maximum at $\mu = 90$ °. Hence, the lamellar fragmentation did not occur in this region. The peak at $\mu = 90$ ° in Figure S15(b) probably reflects the increase in the volume of the lamellar structure associated with the change in $D_{\|}$ (Figure S15(a)). The μ dependences of the diffraction peak intensities from the $(110)_{ortho}$ and $(200)_{ortho}$ planes in Figures S15(c) and (d), respectively, show the orientation of the lattice structure did not change much with the strain. The slight decrease in the intensities at $\mu = 90$ ° indicates that mechanical melting hardly occurred in this region.

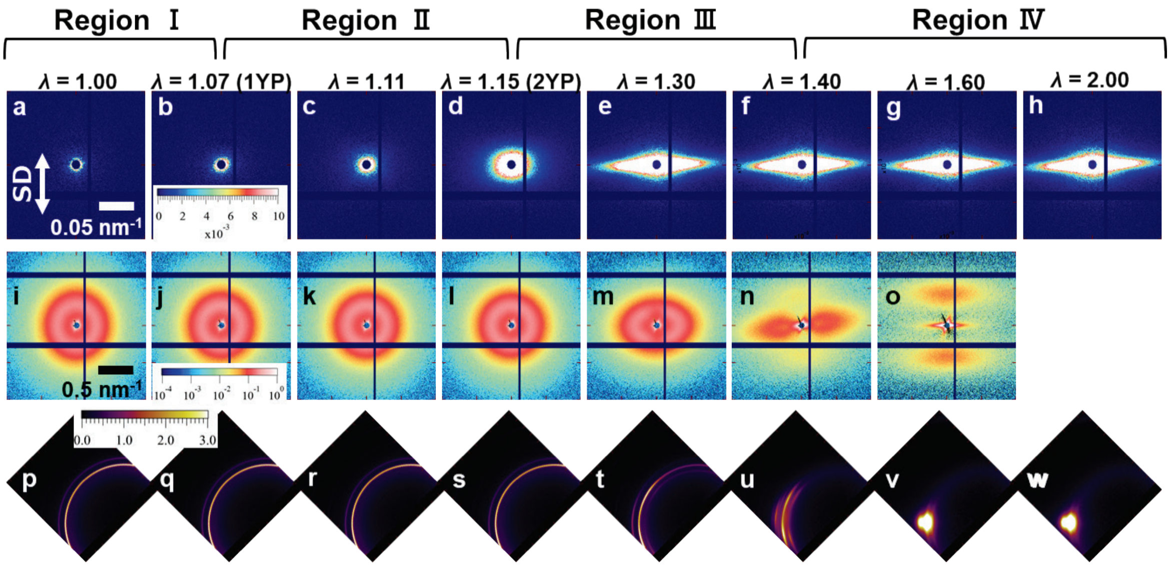

Figure S13 (a)-(h) USAXS, (i)-(p)SAXS, and (q)-(x)WAXS 2D patterns of B-60 with strain. The arrow in part(a) corresponds to the stretching direction.

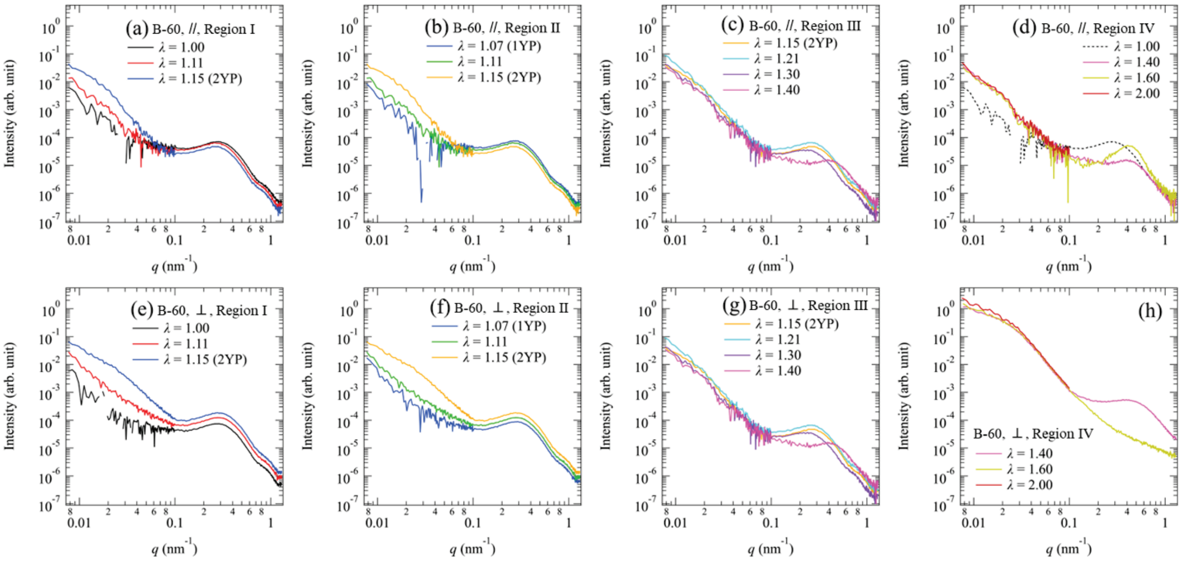

Figure S14 Combined USAXS and SAXS profiles from B-60 in (a) and (e) Region I, (b) and (f) Region II, (c) and (g) Region III, and (d) and (h) Region IV, respectively. The profiles in (a), (b), (c), and (d) and (e), (f), (g), and (h) correspond to the scattering intensities parallel and perpendicular to the stretching directions, respectively.

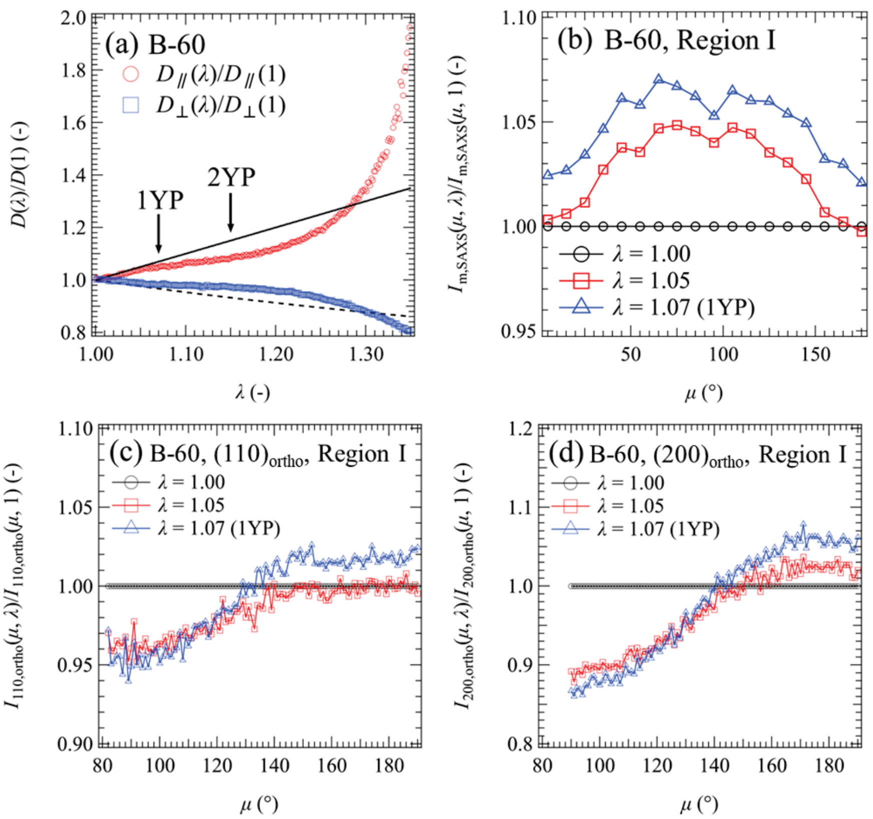

Figure S15 (a) λ dependence of $D_{//}(\lambda)/D_{//}(1)$ and $D_{\perp}(\lambda)/D_{\perp}(1)$ of B-60. The solid and dashed lines are the lines calculated by Eqs. (7) and (8) obeying affine deformation. μ dependence of (b) the reduced peak intensity $I_{m,SAXS}(\mu, \lambda)/I_{m,SAXS}(\mu, 1)$ of SAXS, (c) $I_{110,ortho}(\mu, \lambda)/I_{110,ortho}(\mu, 1)$, and (d) $I_{200,ortho}(\mu, \lambda)/I_{200,ortho}(\mu, 1)$ in WAXS patterns in Region I in B-60.

Region II

In Region II (1.07 (1YP) < λ < 1.15 (2YP)), The little change in the SAXS patterns with strain was observed in Figures S13(k) and (l). The profiles in Figure S14(b) and (f) did not show the change clearly. The peak position did not shift with strain (Figure S15(a)) and the deformation of the lamellar structure did not follow the affine deformation. Contrary to the case of MP-50 and B-50 with lower crystallinity, the deformation was much suppressed. Figure S16(a) showed that lamellar fragmentation started and slowly proceeded in this region. The diffraction peak intensities from the $(110)_{ortho}$ and $(200)_{ortho}$ planes in Figures S16(b) and (c) did not change much with the strain. On the other hand, the patterns were elongated to the direction perpendicular to the stretching direction. The orientation of the scattering pattern was different from those observed in MP-60 and B-50, which contributed to suppressing the enhancement. The increase in the intensity in the USAXS pattern was observed in Figure S13(c) and (d). We think that the voids in B-60 appeared at the defects in the sample and were elongated with strain.

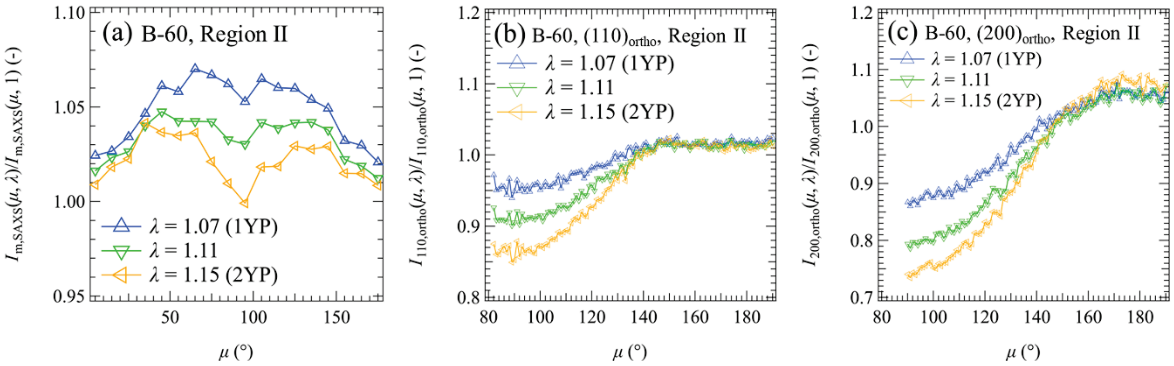

Figure S16 μ dependence of (a) the reduced peak intensity $I_{m,SAXS}(\mu, \lambda)/I_{m,SAXS}(\mu, 1)$ of SAXS patterns, (b) $I_{110,ortho}(\mu, \lambda)/I_{110,ortho}(\mu, 1)$, and (c) $I_{200,ortho}(\mu, \lambda)/I_{200,ortho}(\mu, 1)$ in WAXS patterns in Region II in B-60.

Region III

In Region III or 2YP or at $1.15 < \lambda < 1.40$, the voids were strongly elongated to the stretching direction and a chevron-type morphology was developed. In the USAXS patterns (Figures S13(d)-(f)), the elongated patterns became the streak patterns. The intensity increased drastically, indicating that the elongated voids were formed in this region. On the scale of the lamellar structures (Figures S13(l)-(n)), we observed diagonal characteristic patterns referred to as four-point patterns, attributing to the lamellar fragmentation and rotation. Figures S17(a) makes it clear the formation of the four-point pattern. The diagonal orientation of the $(110)_{ortho}$ and $(010)_{mono}$ peaks in the WAXS patterns (Figures 13(t)-(v) and Figures S17(b) and (d)) supports the chevron-type morphology and the increase in the $(200)_{ortho}$ peak oriented to the perpendicular direction reflects the realignment of the melted chains (Figures S17(c)).

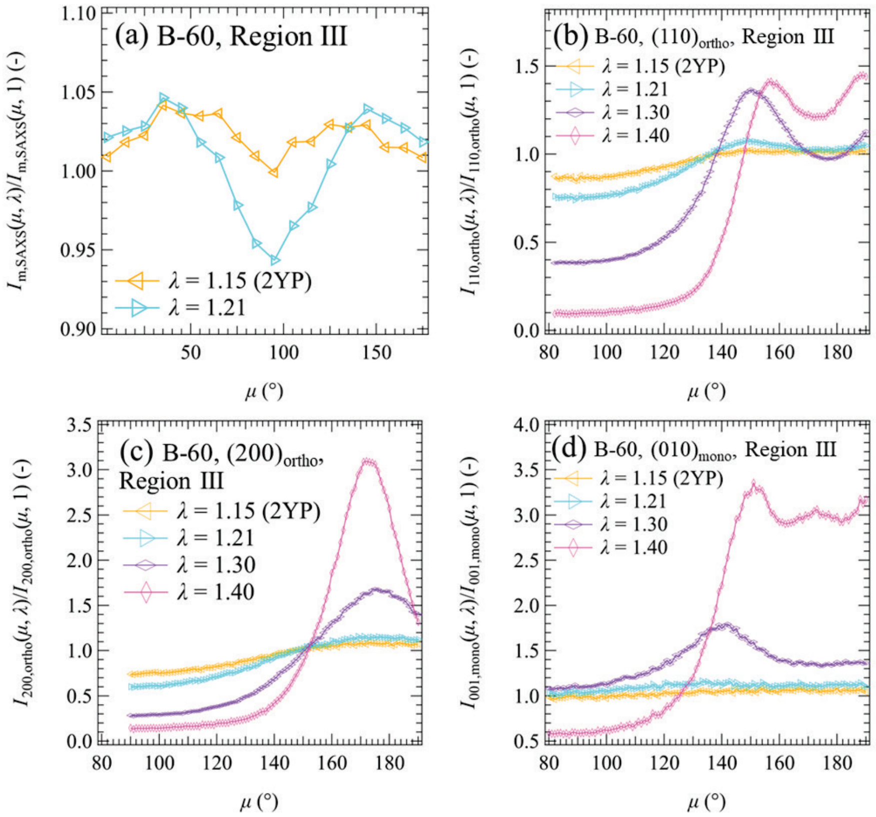

Figure S17 μ dependence of (a) the reduced peak intensity $I_{\mathrm{m,SAXS}}(\mu, \lambda)/I_{\mathrm{m,SAXS}}(\mu, 1)$ of SAXS patterns, (b) $I_{110,\mathrm{ortho}}(\mu, \lambda)/I_{110,\mathrm{ortho}}(\mu, 1)$, (c) $I_{200,\mathrm{ortho}}(\mu, \lambda)/I_{200,\mathrm{ortho}}(\mu, 1)$, and (d) $I_{010,\mathrm{mono}}(\mu, \lambda)/ I_{010,\mathrm{mono}}(\mu, 1)$ in WAXS patterns in Region III in B-60.

Region IV

In Region IV or $1.40 < \lambda$, the void grew with strain on the submicron scale and the chevron-type morphology transformed into a fibrillar structure. The streak patterns in the USAXS (Figures S13(f)-(h)) increased with strain, reflecting the growth of the voids. In the SAXS patterns (Figures S13(n) and (o)), we observed the gradual fading of the four-point pattern and the appearance of the broad peaks at approximately $q = 0.4$ nm^{-1} in the parallel direction, indicating the formation of the fibrillar structure. As shown in the WAXS patterns (Figures S13(u)-(w)) and μ dependences of the diffraction peaks (Figures S18(a) and (c)), the $(110)_{\mathrm{ortho}}$ and $(010)_{\mathrm{mono}}$ peaks corresponding to the four-point pattern decreased while the peak intensity at $\mu = 180$ ° increased with strain. The μ dependence of the $(200)_{\mathrm{ortho}}$ peak (Figure S18(b)) also shows that the intensity at $\mu = 180$ ° increased with strain. These changes also support the formation of a fibrillar structure.

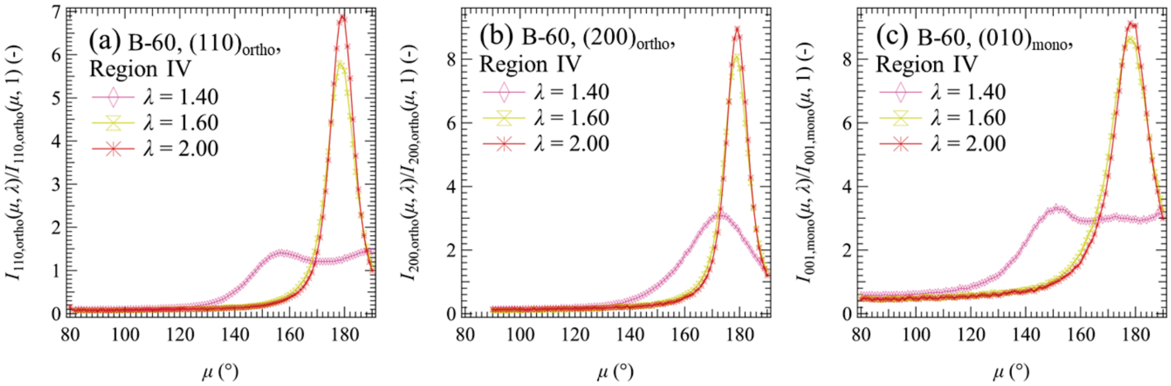

Figure S18 μ dependence of (a) $I_{110,\mathrm{ortho}}(\mu, \lambda)/I_{110,\mathrm{ortho}}(\mu, 1)$, (b) $I_{200,\mathrm{ortho}}(\mu, \lambda)/I_{200,\mathrm{ortho}}(\mu, 1)$, and (c) $I_{010,\mathrm{mono}}(\mu, \lambda)/I_{010,\mathrm{mono}}(\mu, 1)$ in WAXS patterns in Region IV in B-60.
